# Phubbing Behaviour: A Bibliometric Analysis of Scientific Production †

**DOI:** 10.3390/bs15060745

**Published:** 2025-05-28

**Authors:** Ana Cebollero-Salinas, Begoña Gutiérrez-Nieto, Jacobo Cano-Escoriaza

**Affiliations:** 1 Department of Educational Science, Faculty of Education, Universidad de Zaragoza, 50009 Zaragoza, Spain; 2 Department of Accounting and Finance, Universidad de Zaragoza, 50009 Zaragoza, Spain; bgn@unizar.es; 3 Department of Sciences Education, Universidad de Zaragoza, 50009 Zaragoza, Spain; jcano@unizar.es

**Keywords:** literature review, bibliometrics, scientometrics, phubbing, technoference

## Abstract

Phubbing refers to the act of ignoring someone in a face-to-face conversation by paying more attention to a mobile phone. This practice, although common, has been identified as harmful and deserves to be considered a problem. As a result, it has captured the attention of the scientific community, with a significant increase in studies in recent years. However, there is a lack of bibliometric analyses examining existing research on this topic, which would be useful in guiding future studies. This article seeks to fill that gap by providing a detailed bibliometric analysis of phubbing. It analyses the entire production in the Web of Science database between 1985 and 2022 (199 articles and 82 keywords). The study employs citation, co-occurrence, and co-citation analysis techniques using scientific maps created with VOSviewer software. The results indicate that most research has focused on how phubbing affects romantic relationships, with less emphasis on other types of relationships such as family, work, or friends. Areas that require further research are identified, such as motivations for internet use, the tendency to compare oneself on social networks, impulsivity, and the influence of executive functions on phubbing. Further exploration of the relationship between phubbing and other disorders is also suggested. This analysis will serve as a guide and stimulus for future research, offering valuable resources for professionals in psychology, health, and education.

## 1. Introduction

The proliferation of diverse technological devices and the exponential increase in the utilization of applications for establishing ubiquitous and continuous interpersonal connections with friends, colleagues, clients, and family members, coupled with the accessibility to activities of an educational, recreational, and professional nature (X. Wang et al., 2023), have facilitated the intrusion of technology into face-to-face interactions. This phenomenon has been termed “technoference”, a neologism that fuses the concepts of technology and interference (McDaniel & Coyne, 2016). When interference specifically arises from mobile phones—a common source of “technoference”—it is referred to as “phubbing” (McDaniel & Drouin, 2019). The term “phubbing” is a portmanteau derived from combining “phone” and “snubbing”, and it is widely used to describe the act of prioritizing the use of a mobile phone over engaging in face-to-face social interactions (Karadaǧ et al., 2015; Chotpitayasunondh & Douglas, 2016; Roberts & David, 2016; Vanden Abeele et al., 2019). While both terms—technoference and phubbing—are conceptually related, they are often employed interchangeably in academic discourse (e.g., McDaniel et al., 2018; McDaniel & Drouin, 2019; Shao et al., 2022).

In the past years, there has indeed been a significant surge in research focusing on the social implications of phubbing (e.g., Chotpitayasunondh & Douglas, 2016; McDaniel & Coyne, 2016; Halpern & Katz, 2017; Roberts & David, 2016, 2022; Schokkenbroek et al., 2022; Stockdale et al., 2018; Vaterlaus et al., 2020; Vanden Abeele et al., 2019). This interest arises from concerns in the public domain about the harmful effect of phone use during social interactions (Ayar & Gürkan, 2022; Halpern & Katz, 2017; Nuñez & Radtke, 2023; Tandon et al., 2022; van der Schyff et al., 2022; Vanden Abeele et al., 2020; Yang et al., 2023; Zhang et al., 2021) and from the fact that the person suffering phubbing (phubee) perceives it as something negative. Some studies have shown that phubbees feel devalued by the phubber (Vanden Abeele & Postma-Nilsenova, 2018), perceiving them as annoying and disrespectful (Al-Saggaf & O’Donnell, 2019) and feeling disappointed, frustrated (Cebollero-Salinas et al., 2022a) and excluded (Chotpitayasunondh & Douglas, 2018; Knausenberger et al., 2022; McDaniel et al., 2018). Hence, the scientific research on this phenomenon has grown exponentially. In light of the burgeoning interest in the etiology and ramifications of phubbing, there remains a paucity of comprehensive review articles that could serve as a robust foundation for future scholarly endeavors. Within the realm of literature review methodologies, bibliometric quantitative tools emerge as a valuable approach, employing statistical techniques to analyze research topics through the lens of bibliographic resources. These tools offer a systematic and objective means of synthesizing the existing body of knowledge, thereby illuminating trends, gaps, and potential avenues for further investigation in the field of phubbing research (Zupic & Čater, 2015). This article aims to provide a comprehensive bibliometric analysis of phubbing. The combination of these methods with previous qualitative analyses, such as Afdal et al. (2019), Al-Saggaf and O’Donnell (2019), or Dontre (2021), allows for a comprehensive understanding of the research on phubbing behaviour. The new perspective of this study is based on a citation, co-occurrence, and co-citation approach using scientific maps generated with VOSviewer software (Version 1.6.20).

As part of the bibliometric tools, scientific maps will help monitor the configuration of the phubbing literature network to better understand its structure, evolution, and main participants. These scientific maps will be based on different elements, such as keywords, documents, authors, journals, countries, organisations, or cited references. Following this methodology, this study answers three main research questions:

(1) What are the most influential documents, authors, sources, countries, and organisations?
(2) Which are the most used keywords, and how do they perform?
(3) What is the citation pattern in the literature?

This study contributes to the literature in many ways. It helps to identify the influential aspects: documents, authors, sources, countries, and organisations to identify the citation pattern in the literature, the temporal evolution, and the performance of keywords on phubbing, as well as to propose directions for further research. It is also the first study that addresses this work from the Clarivate Analytics Web of Science and uses the VOSviewer software.

The results show the idea of a solid research topic in which progress has been made in identifying some determinants and in understanding the impact on health and personal, family, and work relationships. The topic also provides an overview of the state of research and helps to identify gaps in order to define important issues in this area for the future.

The remainder of the paper is organised as follows:

Section 2 presents research on phubbing. Section 3 includes the methodological design, data, and bibliometric tools used in the analysis. Section 4 presents the main results of the bibliometric analysis. Finally, Section 5 presents the discussion and conclusions.

## 2. Literature Review

### 2.1. Phubbing Behavior: Explanatory Theories

Phubbing behaviour is seen as usual and normalised (Blanca & Bendayan, 2018; Vanden Abeele et al., 2019), although few papers indicate its prevalence. Davey et al. (2018) specifically mention a prevalence of 49.3% in Polish adolescents. Chotpitayasunondh and Douglas (2016) suggest that phubbing has become acceptable, among other reasons, due to the frequency with which it is observed in others. Three theories have been used to shed light on the reasons that could explain the effect of phubbing. On the one hand, the theory of compensatory Internet use (Kardefelt-Winther, 2014) states that people use the Internet to reduce negative emotions such as boredom or loneliness. Thus, the fact of focusing on the mobile phone and ignoring people who are present could be explained as a way to avoid uninteresting conversations or feeling lonely despite being surrounded by peers. This could explain the relationship between negative emotional states, such as loneliness, boredom, fear of missing out, and the problematic smartphone use that is strongly associated with phubbing (Al-Saggaf, 2021; Blanca & Bendayan, 2018; Chotpitayasunondh & Douglas, 2016; van der Schyff et al., 2022; X. P. Xu et al., 2022). On the other hand, the uses and gratification theory (Ruggiero, 2000) can also provide reasons for phubbing as it does for other behaviours, such as problematic Internet use (Cebollero-Salinas et al., 2021). In other words, phubbing can meet an immediate need or provide a gratification effect for the individual as they focus on the mobile phone to satisfy needs such as curiosity about a notification displayed on their screen or their social networks, escapism from boredom, being entertained by an online game, or staying connected with distant people (Al-Saggaf & O’Donnell, 2019). In this regard, another rewarding force that could also drive phubbing behaviour is media multitasking (Gaeta-González et al., 2025), as it offers a sense of satisfying efficacy. Finally, the displacement theory (X. Wang et al., 2017) can explain how phubbing affects a relationship. This theory states that the time spent on smartphones displaces face-to-face human interactions, which affects the quality of a relationship (Al-Saggaf & O’Donnell, 2019). According to Halpern and Katz (2017), texting reduces the perceived quality of a relationship because of the conflicts that it causes between couples and also because it leads to a lack of intimacy, as the activities on the mobile phone displace the closeness a romantic couple needs.

### 2.2. Contexts Where Phubbing Occurs

The pervasive presence of smartphones and their frequent use during face-to-face interactions across various settings have led to extensive research on phubbing and technoference in diverse social relationships contexts such as romantic relationships, co-parenting, verbal relationships with superiors, and peer relationships.

In particular, a large body of research has focused on phubbing in romantic or dating relationships, with a special emphasis on its effect on the relationship (Frackowiak et al., 2022; Halpern & Katz, 2017; McDaniel & Coyne, 2016; Roberts & David, 2016, 2022; Schokkenbroek et al., 2022; Thomas et al., 2022; Vaterlaus et al., 2020; X. Wang et al., 2017). Another large body of work has focused on the study of phubbing in shared parenting, particularly its effects on family relationships and various aspects related to children. (Denecker et al., 2023; Tacca Huamán et al., 2021; Xie et al., 2019; Zhang et al., 2021; X. Wang et al., 2023; Yang et al., 2023). In particular, some researchers have been concerned with phubbing in the upbringing of children up to the age of 5 (Konrad et al., 2021; Lemish et al., 2020; Sundqvist et al., 2020; Vanden Abeele et al., 2020; M. N. Khan et al., 2022; Roberts & David, 2017, 2022; Yasin et al., 2023; Koc & Caliskan, 2022). In addition, although to a lesser extent, phubbing has been studied in supervisor-employee relationships in the workplace. For instance, when the leader at work, the boss, or the supervisor in an educational context exhibits phubbing behaviour. Phubbing among professionals has also been addressed (Tandon et al., 2022). Finally, other work has focused on phubbing among peers, analysing some related variables such as loneliness, boredom, anxiety, and addiction to social networks (Chu et al., 2021; Tanhan et al., 2023; X. P. Xu et al., 2022; Zhao et al., 2021), neuroticism, fear of missing out (FoMO) (Balta et al., 2020), time spent using social networks, (Blanca & Bendayan, 2018), self-control, depression, Internet addiction (Davey et al., 2018), social isolation and coping strategies (Stevic & Matthes, 2023), well-being (Nuñez & Radtke, 2023) as well as personality traits (T’ng et al., 2018). Also, it has been shown that people who engage in phubbing are often negatively affected when they become phubee (Cebollero-Salinas et al., 2022a).

### 2.3. Effects of Phubbing

Over the years, researchers seem to agree that these effects are quite harmful to health and to the quality of personal relationships, including anxiety, depression, excessive use of mobile phones and social networks, increased loneliness, lower level of trust, sense of belonging, and satisfaction in relationships, as well as poorer well-being (Chotpitayasunondh & Douglas, 2018; Roberts & David, 2017; Vanden Abeele et al., 2016). Concerns about the negative impact of phubbing also seem to encompass other effects, such as those of ostracism (Chotpitayasunondh & Douglas, 2018; Knausenberger et al., 2022; McDaniel et al., 2018; Nuñez et al., 2020). In other words, phubbing is used as a means to reject people. More specifically, peer phubbing has also been related to attention-seeking and liking selfies (Hao et al., 2021), smartphone addiction (Lai et al., 2022), social network addiction (X. P. Xu et al., 2022), nomophobia and well-being (Tomczyk & Lizde, 2022), as well as to communication skills (Ayar & Gürkan, 2022). Regarding parental phubbing, there is evidence that it can have an effect on children’s tendency to use their mobile phone in a problematic way (Xie et al., 2019; Zhang et al., 2021; X. Wang et al., 2023), on self-esteem (Tacca Huamán et al., 2021), on a worse perception of family life (Denecker et al., 2023), on depressive symptoms (X. Wang et al., 2020; Yang et al., 2023), children’s cyberbullying (Qu et al., 2022; Stockdale et al., 2018), and on their prosocial behaviour (C. Xu & Xie, 2023). In terms of partner phubbing, there are confirmed effects such as negative mood (McDaniel & Drouin, 2019), lack of relationship satisfaction (Roberts & David, 2016, 2022; Thomas et al., 2022), reduced relationship quality (Halpern & Katz, 2017), the perception of this behaviour (Frackowiak et al., 2022), and digital intrusion (Schokkenbroek et al., 2022), as well as the impact of marital conflict on children’s dependence on mobile phones (Shao et al., 2022). Studies show that trust is undermined, and participation, work performance as well as commitment in the working environment are also reduced (M. N. Khan et al., 2022; Koc & Caliskan, 2022; Roberts & David, 2017; Tandon et al., 2022; T. Xu et al., 2022).

Regarding gender, there are a few papers that have studied whether phubbing is a phenomenon that affects one gender differently from the other. On the one hand, existing studies show a higher prevalence of this phenomenon in girls, both in the adolescent population (Balta et al., 2020; Tomczyk & Lizde, 2022; van der Schyff et al., 2022; X. P. Xu et al., 2022) and in young people and adults (Chotpitayasunondh & Douglas, 2016). On the other hand, some research suggests that the negative connotations of how phubbing is perceived may vary depending on gender. Phubbee girls are more likely to report stronger feelings of social rejection, such as frustration, loneliness, or exclusion, whereas boys only feel annoyed or, at most, disappointed at being phubbed (Cebollero-Salinas et al., 2022a). A few mediating relationships are gender-specific because, as X. P. Xu et al. (2022) indicate, peer phubbing behavior is more related to attention-seeking and liking selfies for boys than for university girls (Hao et al., 2021), and loneliness is a mediator between phubbing and social network addiction to a greater extent in the case of girls.

### 2.4. Determinants of Phubbing

With respect to the question of what determines phubbing behaviour, research has identified several types of predictors. On the one hand, lack of self-control and behavioural addictions such as smartphone addiction, SMS addiction, and media addiction, (Chotpitayasunondh & Douglas, 2016; Davey et al., 2018; Karadaǧ et al., 2015) relationship to Internet addiction (Fernández-Andújar et al., 2022), and relationship to mobile and social network addiction (Aljasir, 2022). On the other hand, personality traits may also predispose people to phubbing. In fact, it has been shown that neuroticism enhances phubbing (Balta et al., 2020), and yet openness is a negative determinant of phubbing behaviour (Erzen et al., 2021; T’ng et al., 2018). Another promising line of work is the analysis of the influence of emotional aspects. Specifically, several authors identify boredom as one of the causes of this behaviour (Al-Saggaf, 2021; Abi Doumit et al., 2023; Gao et al., 2023; Zhao et al., 2021), as well as loneliness (X. P. Xu et al., 2022) and online emotional expression, where emotional e-competences are a protective factor (Gaeta-González et al., 2025). It is noteworthy that the fear of missing out has been the focus of some papers on both adolescents and adults (Blanca & Bendayan, 2018; Chotpitayasunondh & Douglas, 2016; Jabeen et al., 2023; Talan et al., 2023; van der Schyff et al., 2022; Wu & Yang, 2021).

## 3. Empirical Study

This paper aims to analyse the research conducted by the scientific community on phubbing behaviour using a bibliometric approach. Bibliometric studies use statistical analyses of scientific publications (Pritchard, 1969) to obtain objective and unbiased information about a specific research field (Zupic & Čater, 2015). Moral-Muñoz et al. (2020) analyse various software tools for bibliometric analysis, including Bibexcel, Biblioshiny, BiblioMaps, CiteSpace, CitNetExplorer, SciMAT, Sci2Tool, and VOSviewer. The VOSviewer software was chosen for its visualisation quality and the variety of supported data input and output formats. VOSviewer is a powerful tool developed in Java programming language that allows the creation and visualisation of maps based on bibliographic databases. It facilitates the exploration and interpretation of research networks and relationships. The software is freely available for download from www.vosviewer.com (van Eck & Waltman, 2022).

The previous literature has offered reviews of phubbing under qualitative content analysis approaches. Content analysis, as defined by Downe-Wamboldt (1992), is a research method that provides a systematic and objective means to make valid inferences from verbal, visual, or written data in order to describe and quantify specific phenomena, usually by developing categories. Some examples of content analysis on phubbing are Afdal et al. (2019), Al-Saggaf and O’Donnell (2019), and Dontre (2021). Content analysis provides a deeper qualitative understanding of the content and context of publications. Particularly comprehensive in this regard is the analysis by Capilla Garrido et al. (2021), as this study provides a descriptive overview of phubbing up to 2020. In addition, there are some other reviews focusing on a specific context, such as parental phubbing (Braune-Krickau et al., 2021; Mackay et al., 2022; McDaniel, 2019; Tammisalo & Rotkirch, 2022; Zimmerle, 2019).

**Figure 1:**
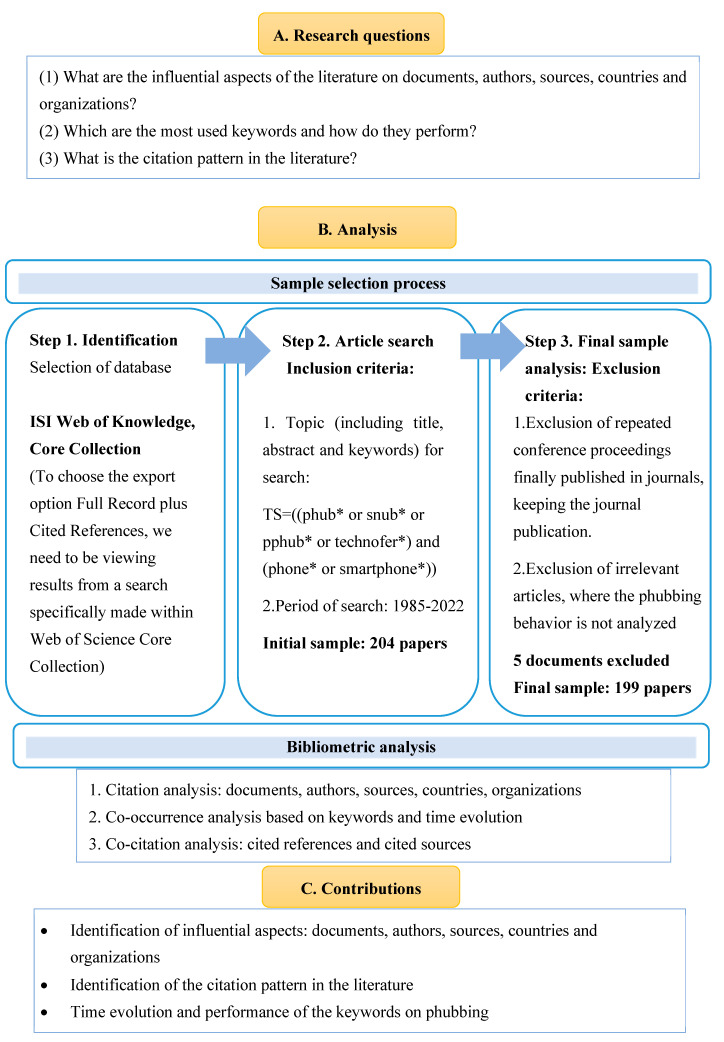
Research design.

However, bibliometric studies involve quantitative analysis of publication and citation patterns. This allows for a numerical and statistical understanding of research trends, productivity, and impact. Bibliometrics allows researchers to identify trends, key contributors, and influential works on a larger scale. Bibliometric data can often be visually represented through charts, graphs, and network maps, aiding in the visualisation of relationships, clusters, and patterns within a research domain. Citation analysis in bibliometrics provides an objective measure of the impact of a publication or an author within the academic community. Bibliometrics can help identify emerging trends by analysing the growth of specific topics or keywords over time. In addition, bibliometric research can deepen the relevance of phubbing behaviour studies along several dimensions, such as journals, countries, terms, organisations, and most cited authors. This approach helps to uncover key connections and relationships between these elements. Figure 1 provides a comprehensive overview of the entire research design of this study, illustrating the research details.

### 3.1. Data

The dataset used in this study was extracted from the Clarivate Analytics Web of Science (WoS) database, widely regarded as a high-quality and comprehensive source. Since its inception in 1950, WoS has amassed a vast collection of top-tier journals, making it a renowned platform for scientific research. Its global recognition and broad coverage of various disciplines allow for meaningful comparisons in scientific domains (Archambault et al., 2006; X. Wang et al., 2017).

Web-based Web of Science was first launched in 1997 and renamed Web of Science Core Collection around 2014 (Liu, 2019). According to Clarivate Analytics (2022), Web of Science Core Collection is the “premier resource on the platform and includes over 21,000 peer-reviewed, high-quality scholarly journals published worldwide (including Open Access journals); over 205,000 conference proceedings; and over 104,000 editorially selected books”.

The search on which this paper is based was conducted on 24 November 2022 in the Web of Science Core Collection. It includes all papers published until that date, downloaded in plaintext format with cited references, to be processed with the VOSviewer software. The search was performed in the field theme (which includes title, abstract and keywords). The primary search term was “phubbing”. To ensure that no relevant studies were missed, the terms “snubbing”, “pphubbing” (referring to partner phubbing), and “technoference” focused on the use of phones were also included. As a result, the comprehensive search strategy was as follows: (phub* OR snub* OR pphub* OR technofer*) AND (phone* OR smartphone*). The use of the asterisk (*) as a Boolean term allowed us to encompass different suffixes and variations of the search terms. In total, the initial sample consisted of 204 papers.

The subsequent stage is to filter the articles according to the inclusion and exclusion criteria, as suggested by Zott et al. (2011). It is described in Figure 1. Two authors independently reviewed the selection process. The final sample consisted of 199 articles for analysis.

### 3.2. Descriptive Analysis

**Figure 2:**
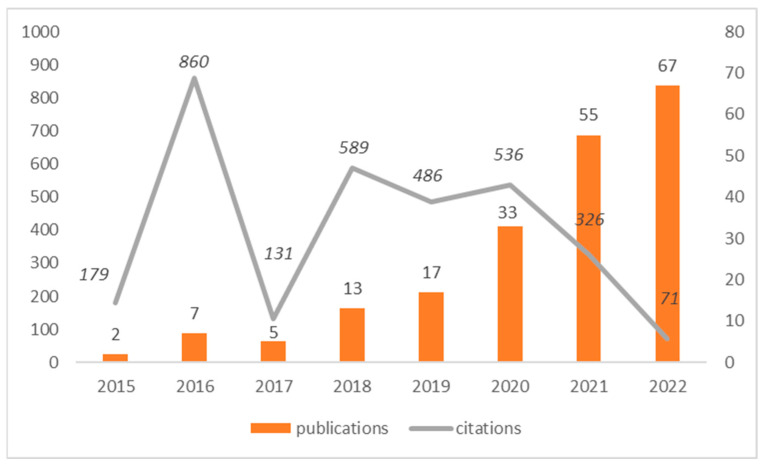
Phubbing: publications and citations (2015–2022).

Figure 2 shows that the earliest paper was published in 2015. The number of publications remains relatively stagnant until 2019 but experiences an exponential increase thereafter. During the 2015–2019 period, the average number of publications per year is less than 10. However, from 2020 to 2022, the average number of publications reaches 51 publications per year. The citation figures report the variable Total Times Cited Count, articles that have cited this parent paper, and articles that were published after this one. Clearly, two articles published in 2016 have a significant influence on subsequent research, as will be verified later.

**Figure 3:**
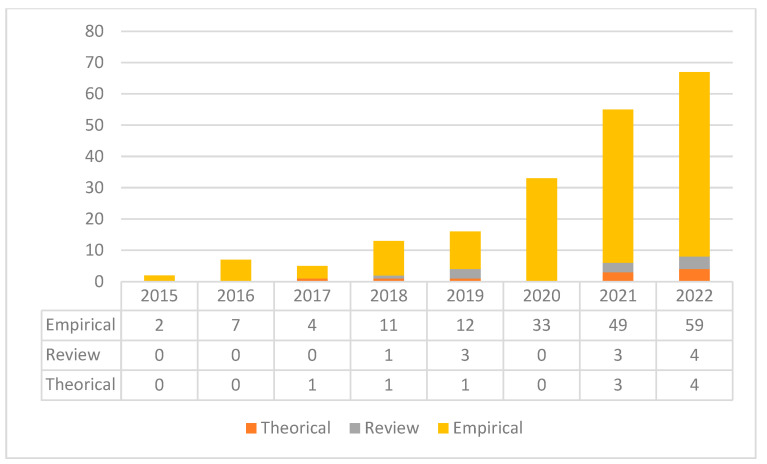
Phubbing: publications and research categories (2015–2022).

Based on the research method, the publications are categorised into three clusters: theoretical, review, and empirical. This classification is illustrated in Figure 3, which highlights the phubbing research as a robust empirical phenomenon throughout the entire analysis period. Most of the publications (89.45%) fall under the category of empirical research. A total of 5.03% of the publications focus on the development of theoretical models, while review contributions make up the remaining 5.53% in this field.

### 3.3. Bibliometric Tool

The VOSviewer software is used to generate knowledge maps highlighting different relevant aspects of the selected 199 papers. Initially, a citation analysis (van Eck & Waltman, 2010) is conducted, focusing on the following items: documents, sources, authors, countries, and organisations. The relationships between these items on the maps are established based on the frequency of mutual citations. To complement the visual interpretation of the maps, the software allows us to download text files containing clusters, weights, and scores associated with the items displayed. These files are used to create the tables in Section 4.1, providing a more comprehensive and detailed representation of the information extracted from the maps.

Additionally, a co-occurrence analysis (Callon et al., 1983) using keywords was conducted. In this analysis, the relatedness of keywords is based on the number of documents in which they occur together (Bevilacqua et al., 2019). The measure of co-occurrence represents the number of publications where both keywords are present simultaneously (van Eck & Waltman, 2017). This analysis allows us to identify patterns of keyword associations and explore the relationships between different concepts within the dataset.

Last, a co-citation analysis (Small, 1973) is performed to investigate the citations within the 199 papers of the sample. This analysis focuses on two elements: cited references and cited sources. The relatedness of these elements is determined by the number of times they are cited together. Gutiérrez-Nieto et al. (2023) highlight that it is crucial to distinguish between citation and co-citation. A citation link signifies a connection between two elements where one cites the other. In contrast, a co-citation link connects two elements that are both cited in the same document.

## 4. Results of the Analysis

### 4.1. Relevance of Documents, Authors, Sources, Countries and Organisations

The papers authored by Roberts and David (2016) and Chotpitayasunondh and Douglas (2016) are undoubtedly considered seminal references in the field of phubbing studies. These two articles have a significantly higher number of citations and a normalised number of citations compared with other studies listed in Table 1, indicating their considerable impact and influence within the research domain.

Brandon McDaniel emerges as the most prolific author in the list of one hundred ninety-nine papers, with nine articles published and the highest figure of normalised number of citations. Following him closely are three authors, in descending order of citation counts, who have contributed eight articles each: Pengcheng Wang, Xingchao Wang, and Li Lei. Table 2, however, presents the list of the top 10 authors working on phubbing based on their impact measured by the number of citations they have received. This table was built using a thesaurus file. As expected, the top positions in this ranking are held by the authors of the two most cited publications, namely Varoth Chotpitayasunondh, Karen Douglas, Meredith David, and Brandon McDaniel. These authors have made significant contributions to the field of phubbing research, as evidenced by their considerable citation counts.

Another indicator is the list of the most prolific journals. Table 1 highlights that two of the five most cited articles are published in *Computers in Human Behavior*, which is also identified as the most productive journal according to Table 3, which presents a list of the top academic journals ranked by the number of published documents. The remarkable impact of *Computers in Human Behavior* is evident in both the number of citations and the normalised number of citations received by its published articles. Furthermore, Table 3 reveals that the top journals listed are responsible for a significant portion, 55.77% to be precise, of the total publications on the topic of phubbing. According to the latest available rankings, the three most prolific journals (*Computers in Human Behavior*, *Children and Youth Services Review*, and *Current Psychology*) are ranked in the average Journal Impact Factor percentiles as 97.2, 79.3, and 62.2, respectively, with percentile 100 as the highest impact factor.

**Figure 4:**
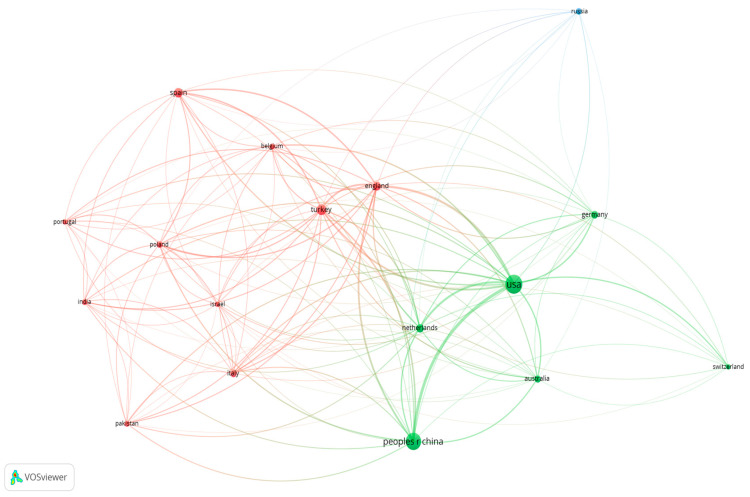
Phubbing: most prolific countries (number of published documents).

Table 4 presents an analysis of the most prolific countries in terms of research publications on phubbing. There are some countries not shown in Table 4 because they have published fewer documents. However, they have received an important number of citations: Belgium, for instance has one hundred ninety-four citations to its six documents. Data of normalised citations confirm this ranking, with USA and China at the top. The variable link between two countries measures the times one country cites the other. Figure 4 also displays a network visualisation of countries to see their relatedness based on the number of times they cite each other (Türkeli et al., 2018). Specifically, in the blue cluster, Russia is more isolated from the rest of the publications; in the green cluster, the USA and China are strongly related to countries such as Australia, The Netherlands, and Germany; and in the red cluster, the UK, Turkey, and Spain are mostly associated with other European countries such as Belgium, the Mediterranean countries and Israel. It is noteworthy that connections between the green and red clusters are abundant, especially between Spain and the USA.

Table 5 offers detailed information regarding the affiliation of authors, focusing on the institutions they belong to. The table is organised first by the number of published documents and second by the number of citations received. The top 10 positions are included in this analysis. According to the normalised citation figures, the top three institutions in the ranking are Chinese: Renmin University, Shanxi University, and Northeast Normal University. The reported institutions of Table 5 also show multiple citation links, where one institution cites the other, a finding similar to the one in Table 4.

### 4.2. Co-Occurrence Network Analysis

The co-occurrence analyses are valuable when identifying the most relevant keywords within the research on phubbing. The visualisation of the mapping provides a comprehensive overview of the relationships between keywords and the formation of clusters. This view helps identify distinct clusters of related topics, allowing researchers to gain insights into the main research directions within the study of phubbing.

The program selects 108 keywords with a minimum number of five occurrences. The sample is then manually refined using a thesaurus file, which is useful for ignoring general keywords (e.g., model or author), merging synonyms (e.g., mobile phone and smartphone), and correcting spelling differences (e.g., phones and phone). Finally, the program selects 82 keywords, the ones with the greatest total link strength with other keywords. According to van Eck and Waltman (2022), each link has a strength, represented by a positive numerical value. The higher this value, the stronger the link (Melega, 2025). The total link strength means the number of publications in which two keywords occur together.

**Figure 5:**
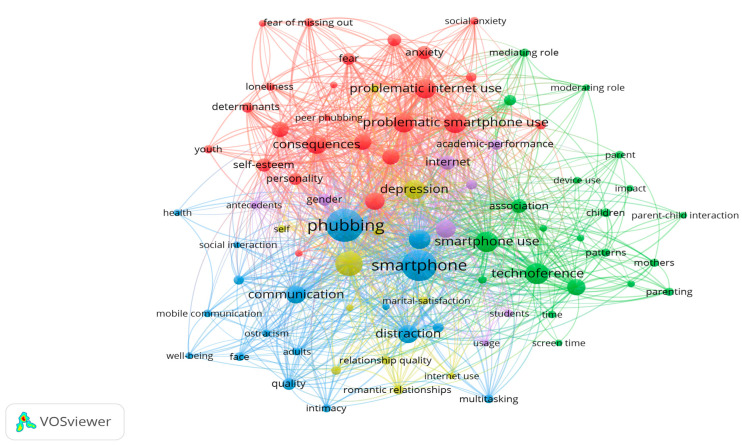
Phubbing: network visualisation of co-occurring keywords.

The software uses distance-based mapping techniques to generate a network visualisation of the co-occurring keywords, as presented in Figure 5. The closeness between keywords is proportional to the number of documents in which they occur together, and the size of the node is proportional to their occurrence. There are five representative clusters with at least five keywords.

The map has multiple connections since the topic has been addressed in different areas and from different perspectives. Map groups can be associated with particular subtopics. The green cluster brings together work related to the technoference node. It is logical that the word smartphone use shows the highest number of connections, as it responds to the type of device being studied and causes interference in relationships. As this cluster shows, this term is most commonly used in articles concerning parents, children, and peer relationships. The blue group focuses on how phubbing is related to effects on communication such as distraction, intimacy, quality of social interaction, ostracism, and thus well-being. The yellow cluster includes a specific line of research focused on depressive symptoms in romantic relationships. The red cluster includes studies on the determinants and consequences of phubbing, highlighting how it is related to the problematic use of mobile phones or the Internet, anxiety, personality, self-esteem, FoMO, and loneliness. Finally, the purple cluster includes papers related to gender, either with respect to the background or to the groups in which it has been analysed, such as students.

### 4.3. Co-Citation Analysis

**Figure 6:**
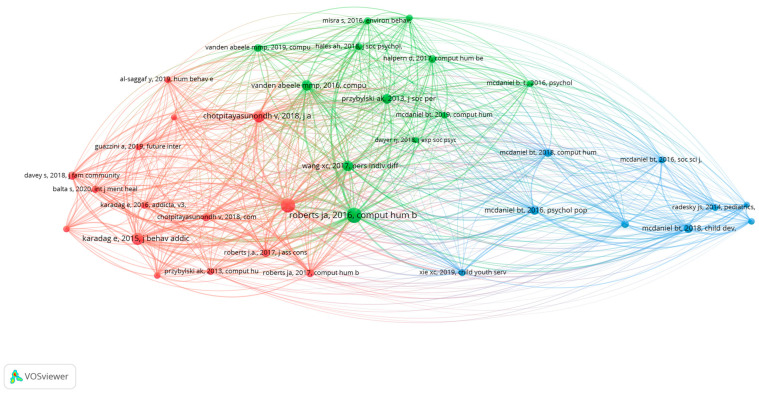
Phubbing: network visualisation of co-citation (cited references).

The co-citation analysis is a valuable method to assess the foundations of the phubbing research, by examining the patterns of citations within the literature. In the first analysis (Figure 6), the analysis units are the cited references of the papers of the sample, where the relatedness of the references in the map is determined by the number of times they are cited together in the same document. The size of the node is proportional to the number of citations (Spatti et al., 2022). To perform the count, the software offers the possibility of choosing between full counting and fractional counting. Fractional counting reduces the influence of documents with a large number of authors. Furthermore, according to Perianes-Rodriguez et al. (2016), the fractional counting approach is preferable to the full counting one.

Figure 6 shows various groupings or clusters. On the one hand, the blue cluster focuses on authors with several publications on phubbing in romantic relationships and parental phubbing. Almost all of them are based on publications by B.T. McDaniel. The green cluster brings together authors who have published articles on partner phubbing and the effects on the quality of interactions in romantic relationships. Finally, the red group includes the authors who have analysed the perceptions, predictors, impacts, and consequences of phubbing.

**Figure 7:**
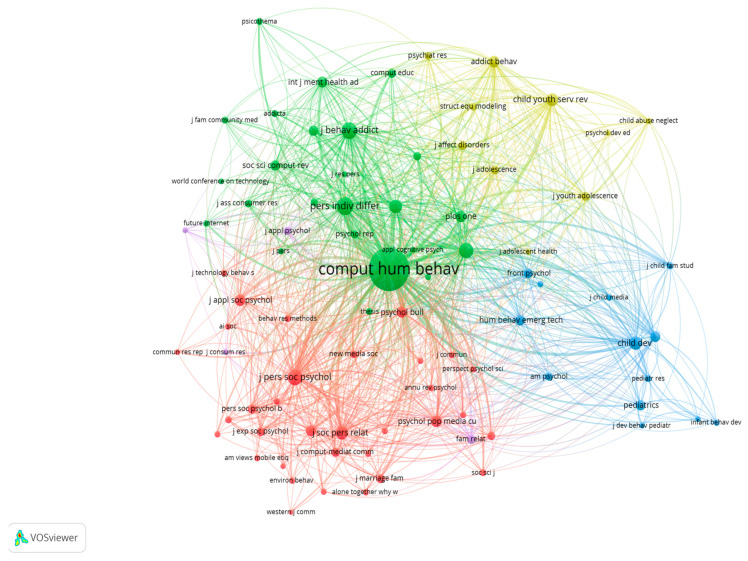
Phubbing: network visualisation of co-citation (cited sources).

Figure 7 represents the network considering the cited sources of the papers of the sample as the unit of analysis, where the relatedness of the journals in the map is determined by the number of times they are cited together in the same document. The size of the node is proportional to the number of citations (Spatti et al., 2022).

Thus, in Figure 7, the cluster that stands out the most is the green one, especially the journal *Computers in Human Behavior*. Not surprisingly, it is the journal that has published the most articles on phubbing. This journal, along with the vast majority of this cluster, is categorised within WoS in the fields of Multidisciplinary Psychology, Experimental Psychology, Computer Science, Psychiatry, and Substance Abuse. The journals included in the blue cluster are especially focused on the fields of Communication, Interdisciplinary Social Sciences, and Paediatrics. The yellow cluster includes Clinical Neurology, Psychiatry, Clinical Psychology, Substance Abuse, Family Studies, and Social Work, and the red cluster includes Social Psychology, Computer Science, Interdisciplinary Applications, Education, and Educational Research and Communication. Considering several categories together, the yellow and blue clusters focus on journals about children and youth (e.g., *Italian Journal of Paediatrics*, *Child and Youth Services Review*, and *Child Development Perspectives*). The green cluster includes a good number of journals that are more oriented toward computer science and human interactions with computers (e.g., *Computers and Human Behavior*, *Social Science Computer Review*, and *Future Internet*), and the red cluster includes journals around social and group relationships, such as *Journal of Social and Personal Relationships* and *The Journal of Social Psychology*.

**Figure 8:**
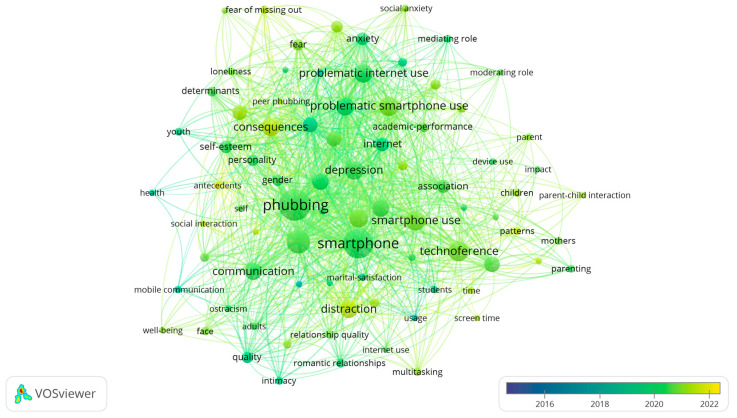
Phubbing: temporal overlay visualisation of co-occurring keywords in publications.

Both Figure 8 and Figure 5 analyse the co-occurring keywords that appear in the phubbing publications. However, while Figure 5 displays the clusters of keywords, Figure 8 illustrates the evolution over time using an overlay visualisation of a map, where keywords are coloured according to a specific score; in this case, the years.

There are hardly any dark blue nodes, as between the years 2015 and 2018, the average number of articles was very modest. However, there are more dark green nodes (years 2019–2020) as the number of articles on phubbing increases exponentially from that year onwards. The co-occurring keywords focus on the analysis of phubbing in relation to some of its predictors and consequences, such as ostracism, anxiety, problematic use of smartphones, relationship quality, intimacy, and satisfaction in student relationships, but especially in romantic and family relationships. The co-occurring keywords around the lighter green emphasise some health consequences such as depression, stress, scale validation, peer phubbing, and parental phubbing. Finally, in yellow, the keywords focus on predictors and impacts of phubbing, such as FoMO, screen time, distraction, and well-being.

## 5. Discussion and Conclusions

Phubbing has become a behaviour that has captured the interest of researchers worldwide, with publications on the phenomenon growing exponentially. In the absence of papers that offer a comprehensive overview of the research to date, the aim of this work is to provide a complete bibliometric analysis of phubbing. This is the first study based on a citation, co-occurrence, and co-citation approach using scientific maps generated with VOSviewer software.

Concerning the first research question, this analysis examines how the number of phubbing articles has increased from its beginning until 2022. Research on the subject took off in 2020, both in theoretical and review articles, but especially in empirical articles. In them, explanatory theories have been suggested, and a few of its determinants have been confirmed in a variety of samples such as in Balta et al. (2020); Davey et al. (2018); Gao et al. (2023); Karadaǧ et al. (2015); Xie et al. (2019). In addition, some of its impacts have also been corroborated (Chotpitayasunondh & Douglas, 2018; Nuñez & Radtke, 2023; Roberts & David, 2017; Vanden Abeele et al., 2016), so it can be concluded that phubbing is an emerging but solid research phenomenon. On the other hand, the origin of the research shows the global interest in this phenomenon, both in the USA and in many European countries, as well as in China, Turkey, and Australia. Similarly, the analysis identifies the main journals dealing with this research topic, not only by the frequency of publication but also by the citations they receive, highlighting the psychological sources.

Answering the second and third research questions of our study, the co-occurring keywords and co-citation analyses carried out have resulted in a map of the relationships of both the keywords and the citations of the different articles. It can be concluded that phubbing affects all areas of relationships and that this behaviour has been studied primarily in romantic relationships and, less recently, in family, work, and peer relationships. In addition, the results show that research on phubbing has focused on a few predictors, especially personality traits, addictions, and some emotions such as loneliness and FoMO (e.g., T’ng et al., 2018; X. P. Xu et al., 2022; Zhao et al., 2021) which could explain the use of the internet and phubbing to compensate for these negative states as indicated by the theory of compensatory internet (Kardefelt-Winther, 2014). On the other hand, the effects identified so far are on health (Gao et al., 2023; X. Wang et al., 2017), the quality of social bonds between peers, as well as on personal (Roberts & David, 2017; Vanden Abeele et al., 2016), work engagement and trust bonds (M. N. Khan et al., 2022; Koc & Caliskan, 2022) and a group of research stands out that identifies that the impact of parental phubbing on children’s behaviour is particularly relevant (e.g., Xie et al., 2019; Zhang et al., 2021) which appears to be explained by displacement theory (X. Wang et al., 2017) thus prioritising face-to-face relationships over virtual ones. These lines of work are also corroborated by the analysis of source co-citation, which shows that phubbing arouses interest in journals in the fields of Psychology, Computer Science, Health, Communication, and, to a lesser extent, education, and family journals.

### 5.1. Implications for Researchers: Future Research

Thus, the analyses carried out have identified a number of areas for further research. Although there are theories that can explain the reasons for phubbing, there are no studies that analyse, from a sociological perspective, the reasons why people engage in this behaviour and their relationship with the determinants which have already been discovered. Moreover, factors such as the gratifications sought through Internet use, the inclination toward social comparison on social media, impulsivity, and the role of executive functions remain unexplored variables. In this sense, in recent years, some studies have been published that identify lack of sleep as a new determinant of partner phubbing (Dikere & Turkasian, 2024).

Another line of work to be developed would be how phubbing relates to disorders other than the most studied addictive behaviours to date, the problematic use of mobile phones and the Internet (Chi et al., 2021; T. Xu et al., 2022). Along these lines, since 2022, research has expanded, linking parental phubbing with Internet Gaming Addiction (Z. Wang et al., 2025) and phubbing with nomophobia and lack of sleep and internet (Guerra Ayala et al., 2025).

It is clear that phubbing behaviour is becoming more widespread, and research to date indicates that there is a need to increase knowledge about it in work relationships and how it is linked, for example, with conditions such as burnout and mobbing. As mentioned above, much of the research focuses on the effects of phubbing and its psychological background (Cebollero-Salinas et al., 2024b). However, a specific area of interest is that of habits for responsible use of phones (Cebollero-Salinas et al., 2022b), promoted in schools and families (Elboj et al., 2023). This would be a line of work to improve digital competence and family supervision. It could reveal effective strategies to deal with phubbing and its negative implications. As the field of phubbing research progresses, gaps in our understanding become increasingly evident, highlighting areas that require additional study. In this sense, there are still few studies that analyse the influence of gender, and so far, they all emphasise that it should be taken into account (Balta et al., 2020; Tomczyk & Lizde, 2022; van der Schyff et al., 2022; X. P. Xu et al., 2022).

### 5.2. Implications for the Prevention of Phubbing

Therefore, the results of this study have practical implications, particularly in identifying areas for future research, both for study and for intervention planning. It seems particularly important to develop studies on educational strategies for the family environment and digital health. Specifically, depending on the determinants found, such as loneliness and loneliness would require alternative leisure programmes to screens, as well as preventing excessive use of the internet because of its close relationship with phubbing by providing spaces and times for digital disconnection depending on the age of use in order to avoid excessive internet use. Another implication in the educational sphere could be to accompany families to reduce the use of mobile phones in front of their children, as it is a key factor for children (C. Xu & Xie, 2023; Yang et al., 2023).

These contributions would be really useful for policymakers, educators, health care providers, and families in order to assess the impact of technology and to incorporate its responsible use into the teaching of digital competencies, for example, through collective intelligence, which has produced such good results (Cebollero-Salinas et al., 2024a; Orejudo et al., 2022). Further lines of work can be focused on studying the responsible use of social networks more closely and on the design of socio-educational policies at an international level that help citizens to have greater socio-emotional competencies within the context of digital citizenship (Gaeta-González et al., 2025).

### 5.3. Limitations and Contributions of the Study

This study is not without limitations, one of which is the restriction of the search to a single database, Web of Science, and the fact that the search ended in 2022. Another is that, in the case of affiliations in terms of country of origin or university, the credentials of the first author do not necessarily accurately reflect the origin of the study. The limitations of the software used are based on the fact that (1) the accuracy of VOSviewer results depends on the quality of underlying data; (2) VOSviewer focuses on visualising quantitative patterns and relationships in data but cannot perform a qualitative analysis, i.e., it does not replace the need for critical interpretation of the results; (3) VOSviewer can only work with data that are available in bibliometric databases and cannot include additional or contextual information that is not present in those data. In summary, VOSviewer is a valuable tool for visualising and analysing bibliometric data, but it should be used with caution and in combination with other approaches and tools to gain a complete understanding of a particular research area or dataset.

Despite these limitations, the results have significant results for the understanding of phubbing research. On the one hand, the research conducted here on phubbing contributes to research on literature review, specifically bibliometric reviews of online behaviours. In addition, it is complemented by an in-depth empirical analysis of citations of documents, authors, journals, countries, and institutions, an analysis of co-occurring keywords, and an analysis of co-citations which, to the authors’ knowledge, have not been applied before in phubbing research. At last, this tool has made it possible to identify trends in phubbing research, and the results of this study can help researchers gain an overview of state of the art, determine the current situation, identify critical points and gaps, and anticipate important issues in this field for the future.

### 5.4. Conclusions

In summary, the rapid growth in the use of technology is leading researchers to focus on how the devices affect people’s overall development. Hence the interest in an emerging but already normalised behaviour such as phubbing (Chotpitayasunondh & Douglas, 2016). This study is the first that employs citation, co-occurrence, and co-citation analysis techniques using scientific maps created with VOSviewer software, and it has allowed us to identify less-researched areas, such as motivations for phubbing, the tendency to compare oneself on social networks, impulsivity, and the influence of executive functions on phubbing. Further exploration of the relationship between phubbing and other disorders is also suggested. This analysis will serve as a guide and stimulus for future research, offering valuable resources for professionals in psychology, health, and education.

## Figures and Tables

**Table 1:** Phubbing: most cited publications.

Title	Authors	Journal	Year	Citations	Normalised Number of Citations
My life has become a major distraction from my cell phone: partner phubbing and relationship satisfaction among romantic partners	Roberts, J.A. and David, M.E.	*Computers in Human Behavior* 54, 134–141	2016	260	2.2414
How “phubbing” becomes the norm: the antecedents and consequences of snubbing via smartphone	Chotpitayasunondh, V and Douglas, K. M.	*Computers in Human Behavior* 63, 9–18	2016	251	2.1638
Determinants of phubbing, which is the sum of many virtual addictions: a structural equation model.	Karadag, Engin; Tosuntas, Sule Betul; Erzen, Evren; Duru, Pinar; Bostan, Nalan; Sahin, Berrak Mizrak; Culha, Ilkay; Babadag, Burcu	*Journal of Behavioral Addictions* 4(2), 60–74.	2015	124	1.5215
“Technoference”: the interference of technology in couple relationships and implications for women’s personal and relational well-being.	McDaniel, B.T. and Coyne, S.M.	*Psychology of Popular Media Culture* 5(1), 85–98	2016	115	0.9914
The effects of “phubbing” on social interaction	Chotpitayasunondh, V. and Douglas, K.M.	*Journal of Applied Social Psychology* 48(6), 304–316	2018	108	2.5389

Note: This table reports the five most cited articles included in the sample up until 24 November 2022 according to the Web of Science Core Collection. Normalised number of citations of a document equals the number of citations of the document divided by the average number of citations of all documents published in the same year and is included in data provided by VOSviewer (Falcão & Roseira, 2022).

**Table 2:** Phubbing: top authors (number of received citations).

Name	Documents	Citations	Normalised Number of Citations	Name	Documents	Citations	Normalised Number of Citations
Chotpitayasunondh, Varoth	3	404	5.7605	Vanden Abeele, Mariek	6	282	6.6417
Douglas, Karen	3	404	5.7605	Coyne, Sarah	4	198	3.6488
David, Meredith	6	352	8.1217	Erzen, Evren	4	174	7.6142
Roberts, James	6	352	8.1217	Babadag, Burcu	2	156	1.7973
McDaniel, Brandon	9	326	16.5326	Bostan, Nalan	2	156	1.7973

Note: This table shows the 10 most cited authors included in the sample up until 24 November 2022 according to the Web of Science Core Collection. Normalised number of citations of an author equals the number of citations of the author divided by the average number of citations of all documents published in the same year and is included in data provided by VOSviewer.

**Table 3:** Phubbing: most prolific journals (number of published documents).

Journal	Documents	Citations	Normalized Number of Citations
*Computers in Human Behavior*	20	1081	27.29
*Children and Youth Services Review*	7	150	9.6178
*Current Psychology*	7	29	5.8319
*Mobile Media & Communication*	7	74	5.8831
*Human Behavior and Emerging Technologies*	6	129	9.7033
*International Journal of Environmental Research and Public Health*	6	97	2.6854
*Frontiers in Psychology*	5	28	4.6814

Note: This table shows the seven most prolific journals included in the sample up until 24 November 2022 according to the Web of Science Core Collection. Normalised number of citations of a journal equals the number of citations of the journal divided by the average number of citations of all documents published in the same year and is included in data provided by VOSviewer.

**Table 4:** Phubbing: most prolific countries (number of published documents).

Country	Documents	Citations	Links	Country	Documents	Citations	Links
USA	58	1151	16	Netherlands	11	351	16
People’s R China	46	420	16	Germany	10	48	16
Turkey	19	325	16	Australia	9	119	16
Spain	15	49	15	Italy	8	152	15
England	13	503	16	Poland	7	133	16

Note: This table shows the 10 most prolific countries included in the sample up until 24 November 2022 according to the Web of Science Core Collection. The variable link between two countries measures the times one country cites the other and is included in data provided by VOSviewer.

**Table 5:** Phubbing: most prolific institutions (number of published documents).

Institution	Documents	Citations	Links	Institution	Documents	Citations	Links
Renmin University of China	12	144	8	Ghent University (Belgium)	6	194	8
Tilburg University (Netherlands)	8	304	10	Northeast Normal University (China)	6	125	9
Illinois State University (USA)	8	225	9	University of Malaga (Spain)	6	28	4
Shanxi University (China)	8	101	8	Beijing Normal University (China)	5	68	8
Baylor University (USA)	6	352	10	Central China Normal University	5	55	7

Note: This table shows the 10 most prolific institutions included in the sample up until 24 November 2022 according to the Web of Science Core Collection. The variable link between two institutions measures the times one institution cites the other and is included in data provided by VOSviewer.

## Data Availability

Data is contained within the article.
